# Intracellular interactions between APOBEC3G, RNA, and HIV-1 Gag: APOBEC3G multimerization is dependent on its association with RNA

**DOI:** 10.1186/1742-4690-6-56

**Published:** 2009-06-04

**Authors:** Yeshitila N Friew, Vitaly Boyko, Wei-Shau Hu, Vinay K Pathak

**Affiliations:** 1 HIV Drug Resistance Program, National Cancer Institute-Frederick, Frederick, Maryland 21702-1201, USA

## Abstract

**Background:**

Host restriction factor APOBEC3G (A3G) blocks human immunodeficiency virus type 1 (HIV-1) replication by G-to-A hypermutation, and by inhibiting DNA synthesis and provirus formation. Previous reports have suggested that A3G is a dimer and its virion incorporation is mediated through interactions with viral or nonviral RNAs and/or HIV-1 Gag. We have now employed a bimolecular fluorescence complementation assay (BiFC) to analyze the intracellular A3G-A3G, A3G-RNA, and A3G-Gag interactions in living cells by reconstitution of yellow fluorescent protein (YFP) from its N- or C-terminal fragments.

**Results:**

The results obtained with catalytic domain 1 and 2 (CD1 and CD2) mutants indicate that A3G-A3G and A3G-Gag multimerization is dependent on an intact CD1 domain, which is required for RNA binding. A mutant HIV-1 Gag that exhibits reduced RNA binding also failed to reconstitute BiFC with wild-type A3G, indicating a requirement for both HIV-1 Gag and A3G to bind to RNA for their multimerization. Addition of a non-specific RNA binding peptide (P22) to the N-terminus of a CD1 mutant of A3G restored BiFC and virion incorporation, but failed to inhibit viral replication, indicating that the mutations in CD1 resulted in additional defects that interfere with A3G's antiviral activity.

**Conclusion:**

These studies establish a robust BiFC assay for analysis of intracellular interactions of A3G with other macromolecules. The results indicate that in vivo A3G is a monomer that forms multimers upon binding to RNA. In addition, we observed weak interactions between wild-type A3G molecules and RNA binding-defective mutants of A3G, which could explain previously described protein-protein interactions between purified A3G molecules.

## Background

Human immunodeficiency virus type 1 (HIV-1) has infected over 33 million people in the world, leading to the AIDS pandemic . Recent discovery of intracellular host restriction factors suggests that HIV-1 must overcome these defenses in order to replicate and cause AIDS [1,2]. A3G, a member of the APOBEC3 family of proteins, is a host restriction factor that potently inhibits the replication of HIV-1 vectors that fail to express a functional Vif protein [1]. In the absence of Vif, A3G deaminates cytidines of the viral minus-strand DNA, resulting in G-to-A hypermutation of the viral genome; additionally, A3G inhibits viral DNA synthesis and provirus formation [3-8]. A3G may also inhibit HIV-1 replication by inducing degradation of the HIV DNA [3]. HIV-1 expresses the Vif protein, which binds to A3G and targets it for proteasomal degradation [9-14].

A3G and other APOBEC3 proteins contain two catalytic domains (CD1 and CD2), with the consensus amino acid sequence H-X-E-X_23–28_-P-C-X_2–4_-C [15,16]. The histidine and cysteine residues coordinate Zn^2+^, and the glutamic acid serves as a proton shuttle in the deamination reaction [15]. Substitutions of the HECC residues in the CD1 or CD2 catalytic domains and characterization of A3G and APOBEC3F (A3F) chimeric proteins have shown that cytidine deaminase activity in A3G and A3F is primarily associated with CD2 [17]. CD2 also confers the sequence specificity for A3G cytidine deamination, which is a CC dinucleotide on the minus-strand DNA (a GG dinucleotide on the plus-strand DNA); deamination of a cytidine in the minus-strand DNA most frequently results in replacement of the first G with A in the plus-strand DNA [3,4,6,17]. The CD1 domain of A3G does not possess cytidine deamination activity but has been implicated in RNA binding and viral encapsidation [17,18]. A3G has been known to form dimers and multimers [15,18-20]. Like other members of the cellular deaminase family, A3G binds RNA in vitro [15,21-24]. Co-immunoprecipitation (co-IP) of A3G molecules that possess different immunological tags is dependent on the presence of RNA, suggesting that their multimerization requires RNA binding [18,22,25]. On the other hand, it has been observed that when A3G is purified it forms multimers, suggesting that A3G may form multimers using protein-protein interactions [23,26,27].

Virion incorporation of A3G is required for its antiviral activity and results in hypermutation of the viral minus-strand cDNA during reverse transcription [3-6,21]. The mechanism by which A3G is incorporated into viral particles has not been fully established. Some studies have concluded that there is direct association between A3G and HIV-1 Gag through the NC domain and a linker sequence from A3G [28-30]. This was suggested by the fact that deletions/mutation in Gag NC substantially reduced the packaging of A3G into virus-like particles. Others, including our group, showed that the presence of viral or nonviral RNA is required for A3G-Gag co-IP [31-35].

To determine the nature of A3G-A3G, A3G-RNA, and A3G-Gag interactions, we developed a bimolecular fluorescence complementation (BiFC) assay that allowed us to analyze the interactions in living cells [36,37]. BiFC is based on the association between nonfluorescent N- and C-terminal fragments (NY and CY) of the monomeric yellow fluorescent protein that results in the reconstitution of YFP and fluorescence. The NY and CY fragments have very low affinity for each other; however, if NY and CY are fused to other proteins that can multimerize, then the association of the fusion proteins can result in BiFC. Thus, interactions between proteins that may physically associate with each other can be studied in the intracellular environment of a living cell. In these studies, we used the BiFC assay to analyze A3G-Gag interactions and observed that while wild-type A3G and Gag can reconstitute fluorescence, RNA binding-defective mutants of Gag or A3G failed to reconstitute fluorescence, indicating that both A3G and Gag need to bind RNA for multimerization. We also used the BiFC assay to analyze A3G-A3G interactions and observed that wild-type A3G proteins can interact to reconstitute fluorescence. Furthermore, wild-type A3G and RNA binding-defective mutants of A3G form multimers with a lower efficiency, suggesting that that RNA binding by one A3G may result in a low affinity interaction with another A3G. These results indicate that A3G molecules multimerize upon binding to RNA and that weak interactions that occur upon RNA binding by one A3G molecule may contribute to the stability of the multimers.

## Results

### Expression and characterization of A3G BiFC constructs

**Figure 1:**
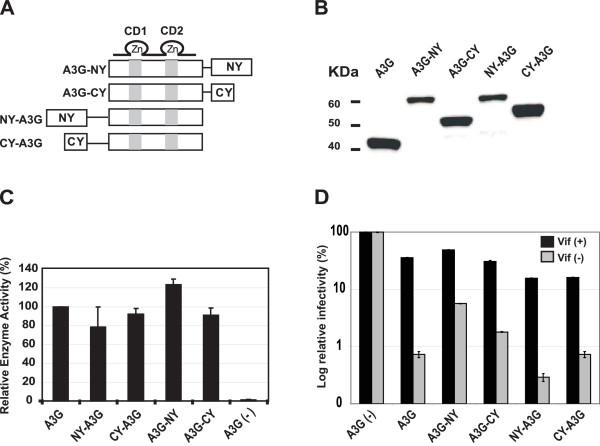
**A3G BiFC constructs and their biological activities**. (A) Structures of A3G BiFC constructs A3G-NY, A3G-CY, NY-A3G, and CY-A3G. The YFP N-terminal (NY) and C-terminal (CY) fragments were fused either to the C-terminal end of A3G (A3G-NY and A3G-CY) or the N-terminal end of A3G (NY-A3G and CY-A3G). The glycine-rich hinge regions (thin lines) for N-terminally tagged BiFC constructs is slightly longer than in the C-terminally tagged constructs. The catalytic domains 1 and 2 (CD1 and CD2) are shown as gray boxes. (B) Western blotting analysis of cells co-transfected with HDV-EGFP along with wild-type A3G or A3G BiFC constructs. The A3G protein was detected using a polyclonal anti-A3G antibody. (C) Relative cytidine deaminase activity in lysates of cells co-transfected with wild-type A3G or A3G BiFC constructs as well as pHDV-EGFP, pC-Help*ΔVif *and pHCMV-G. Total cellular protein (0.3 μg) from each cell lysate was used for determination of enzymatic activity, and the activity in cells transfected with wild-type A3G was set to 100%. Error bars represent the standard error of the mean (s.e.m.) of three independent experiments. (D) Effect of wild-type A3G and A3G BiFC constructs on infectivity of HDV-EGFP. The infectivity of the virions produced in the absence and presence of HIV-1 Vif was determined by flow cytometry analysis of cells infected with the virions. Transfections were also performed in the absence of A3G and Vif, and the proportion of GFP^+ ^cells after infection with HDV-EGFP (23.4% in the absence of Vif, and 28% in the presence of Vif) was set to 100%. Error bars represent the s.e.m. of three independent experiments.

To analyze interactions between A3G and other macromolecules in living cells, we generated a series of A3G BiFC constructs (Fig. 1A). A3G-NY and A3G-CY express A3G that was fused to the NY or CY fragments of YFP at the C-terminus, respectively; A3G and the YFP fragments are separated by a 12 amino acid glycine-rich flexible linker (PGISGGGGGILD). NY-A3G and CY-A3G express A3G that is fused to the NY or CY fragments at the N-terminus, respectively, and the A3G and YFP fragments are separated by a slightly longer 19 amino acid glycine-rich flexible linker (EGITGGGGILDGYLQNSR). We did not determine whether the hinge regions are essential for reconstitution of YFP fluorescence. To evaluate the expression of the A3G BiFC constructs, we implemented Western blot analysis of transiently transfected 293T cells (Fig. 1B). The results showed that all BiFC fusion proteins were expressed; the A3G-NY and NY-A3G proteins were expressed at lower levels than the A3G-CY and CY-A3G proteins. Because of the longer flexible linkers, the NY-A3G and CY-A3G proteins are slightly larger than the A3G-NY and A3G-CY proteins, respectively. To determine whether fusion of NY and CY to A3G affected its cytidine deaminase activity, we prepared lysates of cells transfected with the BiFC constructs and measured the cytidine deaminase activity using a previously described scintillation proximity assay (Fig. 1C). The results showed that cells transfected with all four BiFC constructs had significantly higher levels of cytidine deaminase activity than in the absence of A3G. The enzymatic activities detected were above the linear range of the assay; as a result, differences in expression levels between the fusion proteins were not reflected in the enzymatic activities measured in the cell lysates.

Next, we evaluated whether the A3G BiFC fusion proteins inhibited HIV-1 replication (Fig. 1D). We cotransfected 293T cells with the A3G BiFC constructs in the presence of pHDV-EGFP (an HIV-1 based vector that expresses EGFP), pC-Help*ΔVif *(an HIV-1 helper construct that lacks several cis-acting elements needed for viral replication and expresses all viral genes except Vif, Nef, and Env), and pHCMV-G (a plasmid that expresses vesiculostomatitis virus envelope glycoprotein G). The ability of the virions produced to complete one cycle of replication was determined by infecting 293T cells and analyzing the infected cells for GFP expression by flow cytometry. The proportion of HDV-EGFP infected cells that were GFP-positive in the absence of A3G (23.4% in the absence of Vif and 28% in the presence of Vif) were set to 100%. In the absence of Vif, cotransfection with wild-type A3G or A3G BiFC constructs resulted in severe reductions in GFP^+ ^cells to approximately 2 – 9% of the level observed when cells were infected in the presence of Vif and wild-type A3G (Fig. 1D). These results indicated that the A3G BiFC fusion proteins were able to inhibit HIV-1 replication. In the presence of Vif, the viral infectivity in the presence of wild-type A3G, A3G-NY, and A3G-CY was 31 – 49% of that in the absence of A3G. In the presence of Vif, the viral infectivity in the presence of NY-A3G and CY-A3G was 16%, which was 44% of the wild-type A3G control. This observation suggested that the N-terminally tagged A3G proteins were more resistant to Vif than the wild-type or C-terminally tagged A3G proteins. Nevertheless, all BiFC fusion constructs were sensitive to Vif, since viral infectivity was higher in the presence of Vif compared to the infectivity in the absence of Vif.

### A3G BiFC fusion proteins multimerize and reconstitute fluorescence

**Figure 2:**
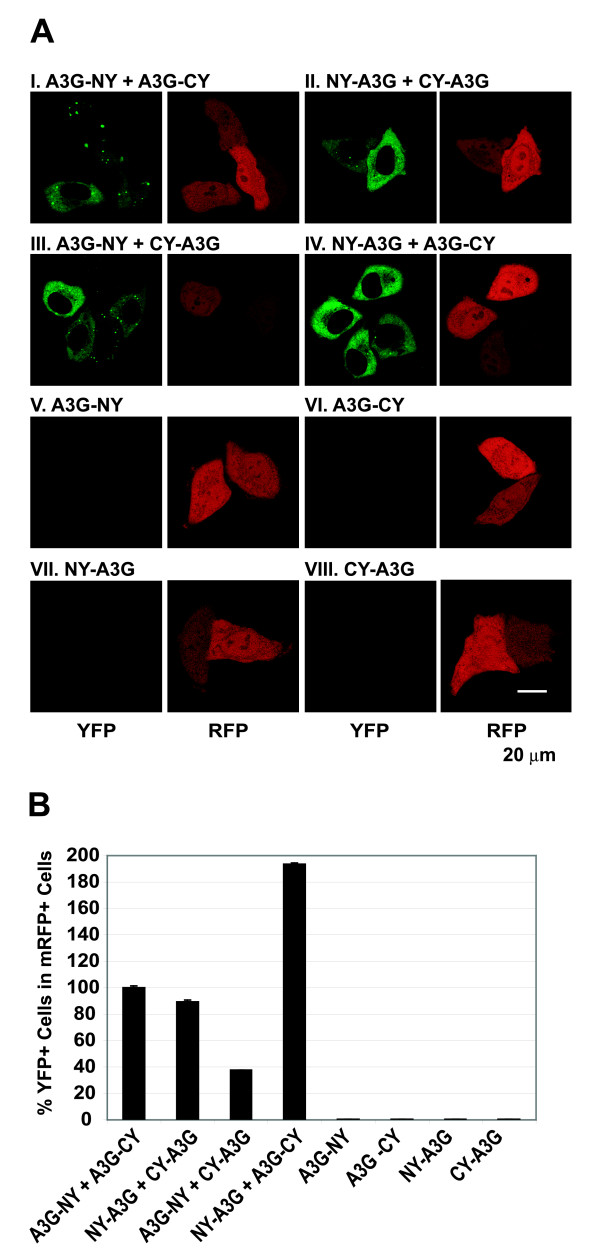
**Reconstitution of YFP fluorescence with A3G BiFC constructs**. (A) Reconstitution of fluorescence upon cotransfection with A3G BiFC constructs. HeLa cells were cotransfected with A3G BiFC constructs and mRFP expression plasmid to identify transfected cells. Fluorescence was reconstituted upon co-transfection with A3G-NY + A3G-CY (I), NY-A3G + CY-A3G (II), A3G-NY + CY-A3G (III), and NY-A3G + A3G-CY (IV). Transfection with A3G-NY (V), A3G-CY (VI), NY-A3G (VII), or CY-A3G (VIII) did not produce YFP fluorescence. (B) Quantfication of BiFC using flow cytometry analysis. 293T cells were co-transfected with A3G BiFC constructs and mRFP expression plasmid as an internal control for transfection, and the percentage of YFP^+ ^cells in mRFP^+ ^cells was determined. The percentage of YFP^+ ^cells in mRFP^+ ^cells after co-transfection with A3G-NY and A3G-CY (15.3%) was set to 100%. The error bars represent the s.e.m. of two independent experiments.

To determine whether the A3G BiFC fusion proteins multimerized in cells and reconstituted fluorescence, we cotransfected HeLa cells with the A3G BiFC constructs in different combinations (Fig. 2A). A mononmeric red fluorescence protein-1 (mRFP1)-expressing plasmid was also cotransfected and served as a control for the identification of transfected cells (Fig. 2A, panels labeled RFP). All BiFC assays to detect reconstitution of YFP fluorescence were performed at 37°C. Cotransfection of A3G-NY and A3G-CY (Fig. 2A-I) as well as NY-A3G and CY-A3G (Fig. 2A-II) reconstituted fluorescence, indicating that the NY and CY fragments that were fused at either the N- or C-terminus of A3G interacted with each other to reconstitute fluorescence (Fig. 2A, panels labeled YFP). Interestingly, cotransfection with A3G-NY and CY-A3G (Fig. 2A-III) as well as NY-A3G and A3G-CY (Fig. 2A-IV) also reconstituted fluorescence, indicating that the NY and CY fragments that were fused to different termini of A3G also interacted to reconstitute fluorescence. As expected, when each A3G BiFC construct was transfected individually, fluorescence was not reconstituted (Figs. 2A-V to 2A-VIII). In addition to diffuse staining throughout the cytoplasm, we also observed aggregations of all A3G fusion proteins (see Fig. 2A, panel I); these aggregates resembled previously described association of A3G with P bodies, but further studies are needed to verify the nature of the aggregations [24,25,38,39]. The appearance of the A3G aggregations in the figures depended on the z-series slice that was used to create the figure, but most cells that showed YFP fluorescence also showed the A3G aggregations.

To determine BiFC efficiency, we performed fluorescence activating cell scanning (FACS) analysis of cells transfected with various BiFC constructs and mRFP expressing plasmid and determined the proportions of YFP^+ ^cells in transfected mRFP+ cells (Fig. 2B). Co-transfection with A3G-NY and A3G-CY reconstituted YFP fluorescence in 15% of the mRFP^+ ^cells (set to 100%). Transfection with NY-A3G and CY-A3G resulted in YFP reconstitution with a similar efficiency (89%). Reconstitution of YFP fluorescence between A3G-NY and CY-A3G was less efficient (37%), whereas YFP fluorescence was reconstituted more efficiently between A3G-CY and NY-A3G (193%). The differences in the efficiency of YFP fluorescence reconstitution may be due to the orientations of the NY and CY fusion proteins in the complexes. When the A3G-NY, A3G-CY, NY-A3G, and CY-A3G constructs were transfected individually, less than 0.1% of the cells expressed YFP fluorescence, indicating that interactions between the NY and CY fragments mediated by A3G were necessary to achieve efficient YFP reconstitution.

### Characterization of A3G BiFC constructs containing CD1 and CD2 mutations

The CD1 of A3G has been shown to be important for RNA binding and virion incorporation [18], whereas CD2 has been shown to possess cytidine deaminase activity [17]. To evaluate the role of CD1 and CD2 in A3G multimerization, we generated a series of BiFC constructs containing mutations in either the CD1 or the CD2. The CD1 residues H65 and C97 were substituted with arginine or serine, respectively, to generate H65R-NY, H65R-CY, C97S-NY, and C97S-CY. Similarly, the CD2 residues H257 and C288 were substituted with arginine and serine, respectively, to generate H257R-NY, H257R-CY, C288S-NY, and C288S-CY.

**Figure 3:**
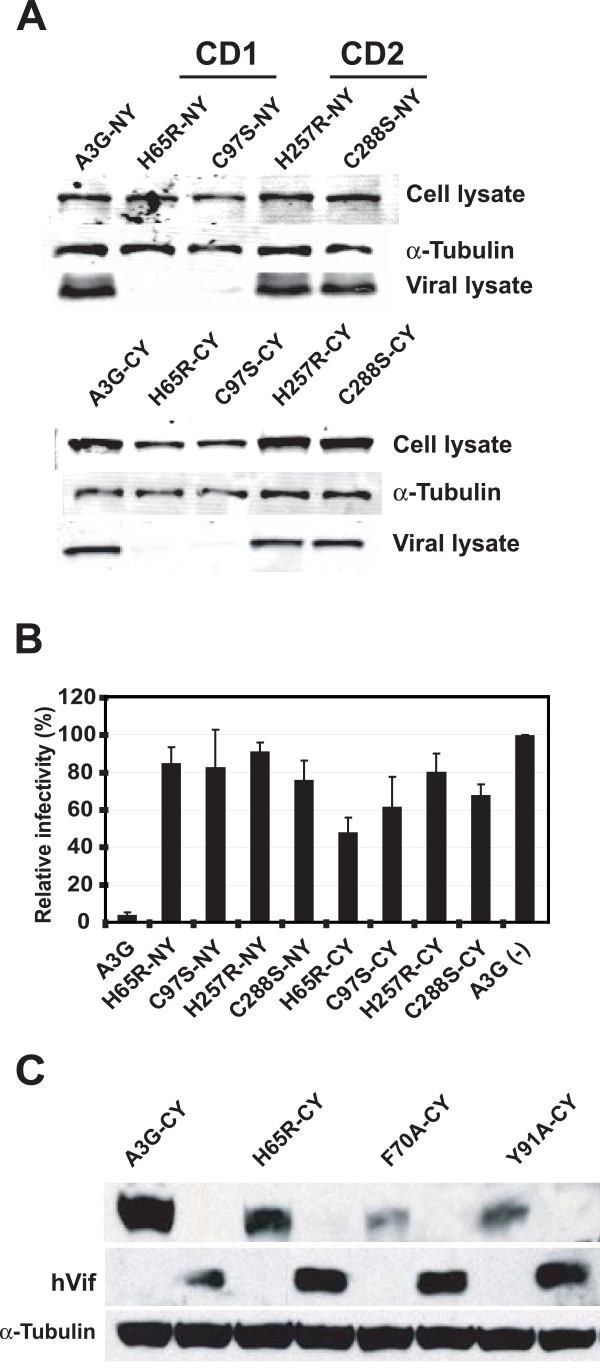
**A3G BiFC constructs containing mutations in CD1 or CD2 and their biological activities**. (A) Western blotting analysis of lysates of 293T cells and viral lysates produced from cells co-transfected with pHDV-EGFP, pC-Help*ΔVif *and pHCMV-G and wild-type A3G or A3G BiFC constructs containing mutations in the CD1 (H65R-NY, H65R-CY, C97S-NY, and C97S-CY) or CD2 (H257R-NY, H257R-CY, C288S-NY, and C288S-CY). The cell lysates were also analyzed using anti-tubulin antibody to insure equivalent loading of cell lysate proteins (panel labeled α-tubulin). (B) Effects of CD1 or CD2 mutations on A3G's ability to inhibit HIV-1 replication. 293T cells were co-transfected with wild-type A3G or A3G BiFC constructs along with pHDV-EGFP, pC-Help*ΔVif*, and pHCMV-G, and the infectivity of the virions produced was determined by flow cytometry analysis of the infected cells for EGFP expression. The proportion of GFP+ cells in the absence of A3G co-transfection was set to 100%. Error bars represent the s.e.m. of three independent experiments. (C) Vif sensitivity of CD1 domain mutants. A3G-CY and CD1 domain mutants H65R-CY, F70A-CY, and Y91A-CY were transfected into 293T cells with and without Vif expression plasmid. A3G fusion proteins were detected by using anti-A3G antibody and HIV-1 Vif was detected using anti-Vif polyclonal antiserum. Anti-tubulin antibody was used to detect tubulin, which served as a loading control.

To determine the effects of the CD1 and CD2 mutations on expression of the A3G BiFC constructs, we transiently transfected 293T cells with the constructs and analyzed the expression of the A3G BiFC fusion proteins by western blotting (Fig. 3A). Preliminary experiments indicated that the A3G BiFC constructs containing the CD1 mutations were expressed at approximately fourfold lower steady-state levels than the wild-type A3G BiFC constructs (data not shown). We therefore transfected fourfold higher amounts of the A3G BiFC constructs containing the CD1 mutations and analyzed the steady-state levels of A3G fusion protein expression. The results showed that after adjusting the amount of plasmid DNA used in the transfection, the levels of the NY fusion proteins were comparable in the cell lysates (Fig. 3A, upper panel). Similarly, the CY fusion proteins were expressed at similar levels in the cell lysates (Fig. 3A, lower panel).

Next, we determined the effects of the CD1 and CD2 mutations on virion incorporation of the A3G BiFC fusion proteins (Fig. 3A). The virions produced from the transfected 293T cells were isolated and equivalent amounts of virions, as determined by p24 capsid (CA) amounts, were analyzed by western blotting. As expected, the results showed that the wild-type A3G and the CD2 mutant BiFC fusion proteins were incorporated into virions, whereas the CD1 mutant BiFC fusion proteins were severely defective in virion incorporation (Fig. 3A, upper and lower panels labeled viral lysate).

Next, we determined whether the CD1 and CD2 mutations influenced the ability of the A3G BiFC constructs to inhibit HIV-1 replication (Fig. 3B). In contrast to wild-type A3G, all of the CD1 and CD2 mutants were severely defective in their ability to inhibit HIV-1 replication. These results are consistent with our observations that the CD1 mutants are defective in virion incorporation (Fig. 3A) and that the CD2 mutants exhibit little or no cytidine deaminase activity (data not shown).

The F70 and Y91 amino acids in the CD1 domain has been previously implicated to be involved in RNA binding [15,18]. We sought to determine whether the RNA-binding defective mutants H65R-CY, F70A-CY, and Y91A-CY are sensitive to Vif binding and proteasomal degradation (Fig. 3C). We cotransfected A3G-CY, H65R-CY, F70A-CY, and Y91A-CY in the presence or absence of pcDNA-hVif, a codon-optimized Vif expression vector [40], and performed western blot analysis. The A3G fusion proteins could be readily detected in the cells in the absence of Vif, but could not be detected in the presence of Vif. The results confirmed that these fusion proteins are sensitive to Vif-mediated proteasomal degradation.

**Figure 4:**
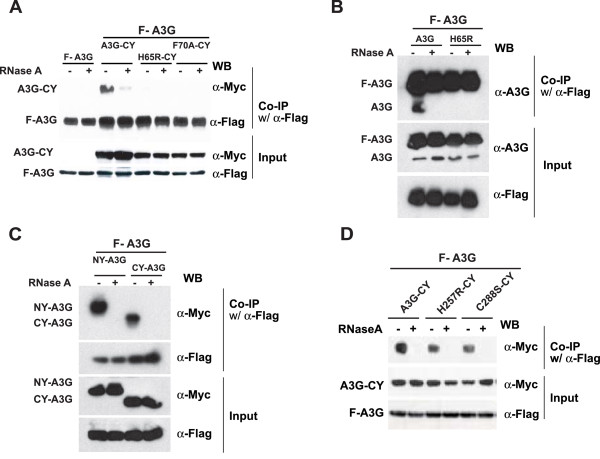
**RNA binding activities of CD1 and CD2 domain mutants of A3G**. (A) Effect of CD1 mutations on the ability of A3G to bind cellular RNA. 293T cells were co-transfected with pF-A3G and empty vector, or pF-A3G and A3G-CY, or pF-A3G and fourfold higher amounts of H65R-CY or F70A-CY DNA compared to pF-A3G. An anti-FLAG antibody was used to co-immunoprecipitate F-A3G and associated proteins in the presence or absence of RNase A treatment. The F-A3G, A3G-CY, H65R-CY, and F70A-CY proteins were detected by Western blot using an anti-A3G antibody. (B) A3G-A3G interactions between F-A3G and untagged A3G proteins. 293T cells were co-transfected with F-A3G and empty vector, F-A3G and fourfold higher amounts of untagged A3G DNA or F-A3G and fourfold higher amounts of untagged H65R mutant of A3G DNA. Co-IP assays were performed as described in Fig. 4A. (C) Effect of N-terminal NY and CY tags on A3G-A3G interactions. 293T cells were co-transfected with F-A3G and NY-A3G or CY-A3G. Co-IP assays were performed as described in Fig. 4A. (D) Effect of CD2 mutations on ability of A3G to bind to RNA. 293T cells were co-transfected with pF-A3G and empty vector, or pF-A3G and A3G-CY, or pF-A3G and fourfold higher amounts of H257R-CY, and C288S-CY DNA compared to pF-A3G. Co-IP assays were performed as described in Fig. 4A.

It has been previously shown that co-IP of A3G proteins tagged with different epitopes is sensitive to RNase A treatment [22,26]. To directly determine the effect of CD1 mutations on RNA binding, we performed co-IP assays from lysates of cells transfected with wild-type A3G that was tagged at the N-terminus with the FLAG epitope (F-A3G) and either H65R-CY or F70A-CY (Fig. 4A). In addition, the co-IP assays were performed before or after treatment of cell lysates with RNase A to degrade cellular RNA. The co-IPs were performed using an anti-FLAG antibody, and the A3G proteins were detected by western blot using an anti-A3G antibody. As expected, A3G-CY was efficiently co-immunoprecipitated in the absence of RNase A treatment; in contrast, upon RNase A treatment, very little A3G-CY was co-immunoprecipitated, indicating that its interaction with F-A3G was mediated through RNA binding. The faint A3G-CY band detected after RNase A treatment is most likely due to incomplete degradation of RNA, since several other co-IP experiments with wild-type A3G proteins did not produce detectable bands after excess RNase A treatment (see Figs. 4B, C, D, and 6B). In contrast to A3G-CY, little or no H65R-CY and F70A-CY were co-immunoprecipitated with F-A3G in the absence of RNase A treatment. The observation indicated that the RNA-dependent interaction between A3G-CY and F-A3G was reduced or eliminated when the H65R or F70A mutation was introduced in the A3G-CY. One likely explanation for the loss of interaction with F-A3G is that the H65R-CY and F70A-CY are defective in RNA binding, which results in a loss of the RNA-dependent interaction.

To determine the possible effects of the C-terminal CY tag on A3G-A3G interactions, we performed co-IP assays with F-A3G and untagged wild-type A3G or untagged H65R mutant A3G (Fig. 4B). The results were identical to those obtained with the A3G-CY and H65R-CY proteins; the wild-type untagged A3G was co-immunoprecipitated with F-A3G in the presence of RNA, but not in the absence of RNA. The untagged H65R mutant A3G was not co-immunoprecipitated with F-A3G in the presence or absence of RNA. To explore the effects of N-terminal tags on A3G-A3G interactions, we co-immunoprecipitated NY-A3G and CY-A3G with F-A3G (Fig. 4C). The result indicated that both of the N-terminally tagged proteins were co-immunoprecipitated with A3G in the presence of RNA but not in the absence of RNA. Thus, similar results obtained with C-terminally tagged, N-terminally tagged, and untagged A3G indicated that A3G-A3G interactions could be detected in the presence of RNA, but the interaction could not be detected if the cell lysates were treated with RNase A to degrade cellular RNA.

We also determined the ability of CD2 mutants H257R-CY and C288S-CY to bind to RNA by performing co-IP assays on lysates of cells transfected with F-A3G and either A3G-CY, H257R-CY, or C288S-CY (Fig. 4D). The results showed that in the absence of RNase A treatment, both H257R-CY and C288S-CY could be co-immunoprecipitated with F-A3G; however, after RNase A treatment, the H257R-CY and C288S-CY could not be co-immunoprecipitated with F-A3G. The result indicated that these CD2 mutants retained their ability to interact with A3G in the presence of RNA.

### Effects of CD1 and CD2 mutations on A3G multimerization

**Figure 5:**
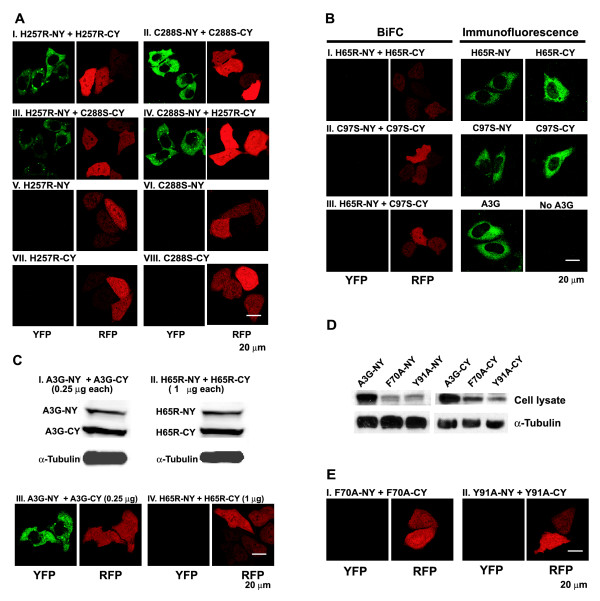
**BiFC assays with CD1 and CD2 mutants of A3G**. (A) BiFC assays with CD2 mutants of A3G. All co-transfections included mRFP expressing plasmid, and RFP expression was used to identify transfected cells (panels labeled RFP). (B) BiFC and immunofluorescence assays with CD1 mutants of A3G. Expression of the CD1 mutants was verified by detection of the H65R-NY, H65R-CY, C97S-NY, and C97S-CY proteins in transfected cells by immunofluorescence. An anti-A3G polyclonal antibody produced in rabbit was used as a primary antibody and Alexa Fluor 568-conjugated goat antibody to rabbit IgG (H+L) (Molecular Probes) was used as secondary fluorescent antibody. (C) Comparison of BiFC and protein expression between WT A3G and H65R mutant A3G. Western blotting analysis of lysates of cells co-transfected with A3G-NY and -CY (I, 0.25 μg DNA each) or H65R-NY and -CY (II, 1 μg DNA each). The A3G proteins were identified by using a polyclonal anti-A3G antibody, and the same lysates were analyzed by using an anti-tubulin antibody to ensure that equivalent amounts were loaded onto gels. (D) Western blotting analysis of lysates of 293T cells and viral lysates produced from cells transfected with CD1 mutants F70A-NY, F70A-CY, Y91A-NY, and Y91A-CY. The cell lysates were also analyzed using anti-tubulin antibody to insure equivalent loading of cell lysate proteins (panel labeled α-tubulin). (E) BiFC assays with CD1 mutants F70A and Y91A.

We sought to determine whether the CD1 and CD2 mutations in the A3G BiFC constructs influenced their ability to form multimers in living cells and reconstitute fluorescence. To determine the effects of the CD2 mutations, we cotransfected HeLa cells with H257R-NY and H257R-CY mutants (Fig. 5A-I) or with C288S-NY and C288S-CY mutants (Fig. 5A-II). In both cases, the CD2 mutants interacted with each other and reconstituted fluorescence. In addition, we also cotransfected H257R-NY and C288S-CY (Fig. 5A-III) or C288S-NY and H257R-CY (Fig. 5A-IV) and observed that the two different CD2 mutants interacted to reconstitute fluorescence. These results indicated that CD2 mutations do not affect the ability of the A3G BiFC fusion proteins to form multimers. Transfection of the individual CD2 mutants tagged with the NY or CY fragment failed to produce fluorescence, indicating that BiFC was required to reconstitute fluorescence (Fig. 5A, panels V – VIII).

We then determined the effects of the CD1 mutations on the ability of the A3G BiFC fusion proteins to interact and reconstitute fluorescence. We cotransfected HeLa cells with H65R-NY and H65R-CY (Fig. 5B-I), C97S-NY and C97S-CY (Fig. 5B-II), or H65R-NY + C97S-CY (Fig. 5B-III). In contrast to the CD2 mutants, the CD1 domain mutants failed to reconstitute fluorescence (Fig. 5B, panels labeled YFP). To verify that the CD1 mutants were expressed in the cotransfected HeLa cells, we performed immunofluorescence studies using an anti-A3G polyclonal antiserum to detect A3G expression (Fig. 5B). The results showed that expression of H65R-NY, H65R-CY, C97S-NY, C97S-CY, and wild-type A3G is readily detectable, and indicated that the absence of YFP fluorescence in cells transfected with these constructs is not due to a lack of expression.

To further verify that lower levels of expression of the CD1 mutants are not responsible for the absence of BiFC-generated YFP fluorescence, we transfected either 0.25 μg each of the control A3G-NY and A3G-CY constructs, or 1.0 μg each of the H65R-NY and H65R-CY constructs (Fig. 5C-I and 5C-II). Western blotting analysis showed that the amounts of A3G-NY and H65R-NY proteins were similar to each other in the transfected cells. Additionally, the amounts of A3G-CY and H65R-CY proteins were similar to each other. Co-transfection of HeLa cells with 0.25 μg each of the A3G-NY and A3G-CY constructs resulted in reconstitution of fluorescence (Fig. 5C-III). However, co-transfection of HeLa cells with 1.0 μg each of the H65R-NY and H65R-CY constructs did not reconstitute fluorescence (Fig. 5C-IV). These results indicated that the absence of fluorescence in cells cotransfected with H65R-NY and H65R-CY is not due to the lower expression levels of the CD1 domain mutants.

The CD1 mutations H65R and C97S alter the amino acids involved in the zinc-binding H-X-E-*x*_23–28_-C*x*_2–4_-C motif; consequently, these mutations could potentially affect the overall structure of the N-terminal CD1 and prevent reconstitution of fluorescence through other effects not involving RNA binding. To address this concern, we generated F70A-NY, F70A-CY, Y91A-NY, and Y91A-CY constructs that had mutations of the aromatic residues F70 and Y91 that were previously implicated as being critical for RNA binding [18,41]. Western blotting analysis of cell lystates indicated that the NY and CY fusion proteins containing these mutations were expressed (Fig. 5D). However, these mutants failed to reconstitute fluorescence (Fig. 5E) and further supported the conclusion that A3G RNA binding is essential for multimerization. The F70A-NY and Y91A-NY reconstituted weak fluorescence with wild-type A3G-CY, indicating that the lower expression level of these mutants was not responsible for the lack of fluorescence (shown in Fig. 10).

### Fusion of RNA-binding peptide to H65R mutant of A3G restores BiFC

**Figure 6:**
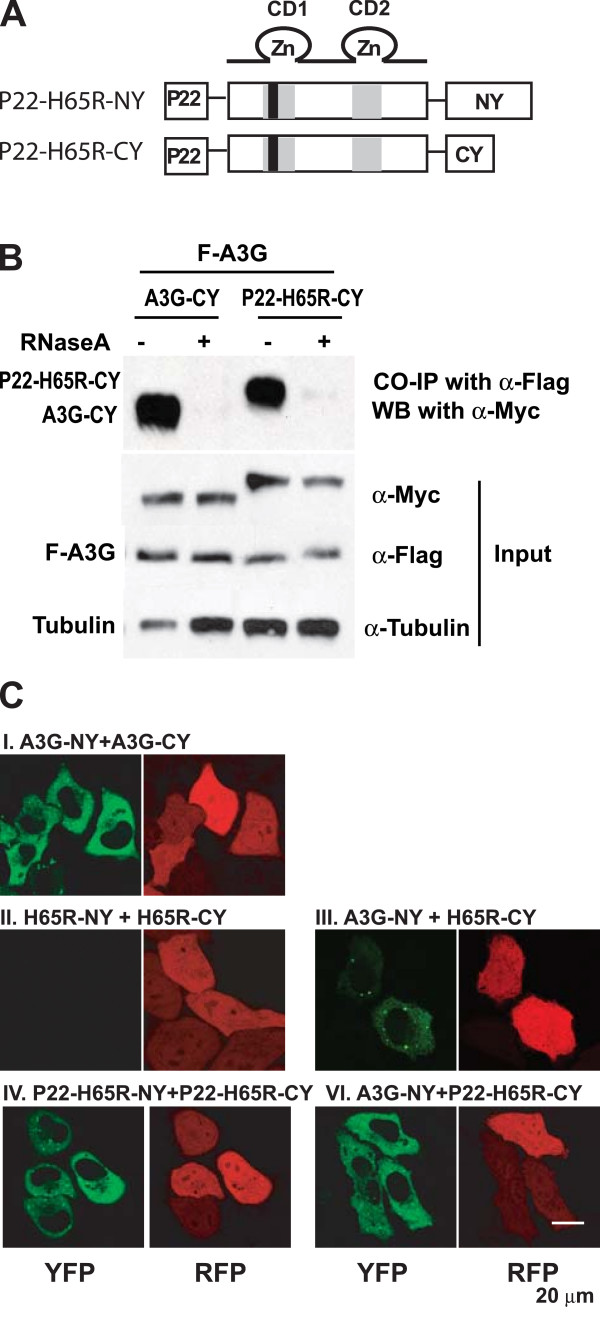
**Effect of non-specific RNA-binding peptide on BiFC with CD1 mutant H65R**. (A) Structure of P22-H65R BiFC constructs. P22 is a 20-amino-acid basic peptide derived from bacteriophage P22 that was fused to the N-terminus of H65R-NY and H65R-CY with a flexible hinge region between P22 and A3G. (B) Effect of P22 peptide on ability of H65R mutant to bind to RNA. 293T cells were co-transfected with pF-A3G and empty vector, or pF-A3G and A3G-CY, or pF-A3G and fourfold higher amount of P22-H65R-CY compared to pF-A3G. Co-IP assays were performed as described for Fig. 4A. (C) BiFC assays to evaluate interactions between wild-type and mutant A3Gs.

To determine whether RNA binding of A3G is sufficient to restore BiFC, we generated expression constructs in which P22, a non-specific RNA binding peptide (GNAKTRRHERRRKLAIERDTIGYS), was inserted between the initiation AUG codon and the second codon of the CD1 mutants H65R-NY and H65R-CY (Fig. 6A). The P22 peptide was derived from the P22 bacteriophage, which specifically associates with a stemloop with high affinity in vitro [42,43]. However, the P22 peptide binds to RNA in a non-specific manner (V. Boyko and W.-S. Hu, unpublished observations).

We determined whether the P22 peptide restored the RNA binding ability of the H65R-CY mutant by performing co-IP assays on lysates of cells cotransfected with F-A3G and either A3G-CY or P22-H65R-CY (Fig. 6B). The results showed that in the absence of RNase A treatment, both A3G-CY and P22-H65R-CY could be co-immunoprecipitated with F-A3G; however, after RNase A treatment, neither A3G-CY nor P22-H65R-CY protein were co-immunoprecipitated with F-A3G. As shown in Fig. 4A, the H65R-CY protein could not be co-immunoprecipitated with F-A3G. The result indicated that addition of the P22 peptide to the H65R mutant restored its RNA-dependent interaction with F-A3G.

Next, we determined whether the presence of the P22 peptide restored the ability of the H65R mutant to multimerize and reconstitute fluorescence (Fig. 6C). As observed earlier, co-transfection of HeLa cells with A3G-NY and A3G-CY reconstituted fluorescence (Fig. 6C-I), while co-transfection with H65R-NY and H65R-CY failed to reconstitute fluorescence (Fig. 6C-II). In contrast to the results obtained with H65R-NY and H65R-CY, co-transfection of HeLa cells with P22-H65R-NY and P22-H65R-CY reconstituted fluorescence (Fig. 6C-III). When we co-transfected A3G-NY and P22-H65R-CY, fluorescence was also restored (Fig. 6C-IV), indicating that protein-protein interactions between the P22 peptides in the fusion proteins were not responsible for reconstitution of fluorescence between P22-H65R-NY and P22-H65R-CY.

**Figure 7:**
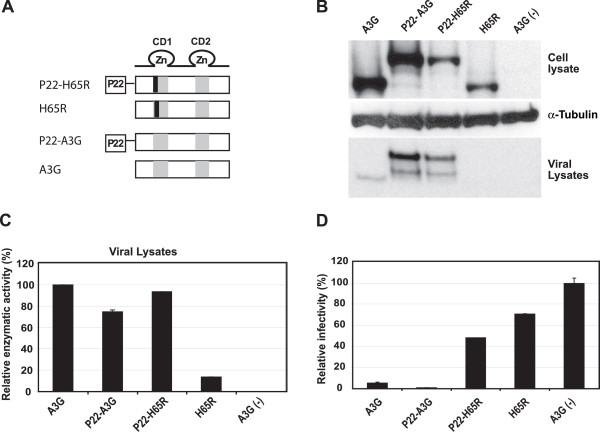
**Effect of non-specific RNA-binding peptide on encapsidation and antiviral activity of CD1 mutant H65R**. (A) Strucures of constructs P22-H65R, H65R, P22-A3G, and wild-type A3G. (B) Western blotting analysis of lysates of 293T cells co-transfected with the A3G constructs as well as pHDV-EGFP, C-Help*ΔVif *and pHCMV-G (panel labeled Cell lysate). The cell lysates were also analyzed using anti-tubulin antibody to insure equivalent loading of cell lysate proteins (panel labeled α-tubulin). Incorporation of A3G BiFC constructs into VLPs (panel labeled Viral lysate) was determined by analyzing viral lysates containing 100 ng of p24 CA. (C) Relative cytidine deaminase activity in viral lysates of virions produced in the presence of wild type A3G, P22-A3G, P22-H65R, H65R, or in the absence of A3G. The cytidine deaminase activity of A3G-CY was set to 100%. (D) Relative HDV-EGFP infectivity in the presence of P22-A3G, P22-H65R and H65R constructs. Infectivity of the virions produced was determined by flow cytometry analysis of cells infected with the virions. The proportion of GFP^+ ^cells after infection with HDV-EGFP in the absence of A3G was set to 100%. Error bars represent the s.e.m. of three independent experiments.

We sought to determine whether the P22 peptide restored virion incorporation of the H65R CD1 mutant. The P22-H65R-NY and P22-H65R-CY constructs were expressed at low levels (data not shown); we therefore generated P22-A3G and P22-H65R, constructs that expressed the P22 peptide at their N-terminus but were not fused to the NY or CY fragments of YFP (Fig. 7A). We then determined the cellular expression and virion incorporation of these constructs (Fig. 7B). The results indicated that the P22-A3G construct was expressed at a level that was similar to untagged A3G, while the P22-H65R and H65R proteins were expressed at lower levels. Analysis of the viral lysates indicated that the P22-A3G was incorporated more efficiently into virions than wild-type A3G. In contrast to the H65R protein, the P22-H65R protein was efficiently incorporated into virions, indicating that the presence of the P22 peptide was sufficient to overcome the virion incorporation defect induced by the H65R mutation.

We sought to determine whether the P22-A3G and P22-H65R proteins were enzymatically active (Fig. 7C). The cytidine deaminase activities in viral lysates containing these proteins were similar to the A3G control, indicating that the presence of the P22 peptide did not interfere with the *in vitro *cytidine deaminase activity. The P22-H65R protein exhibited more cytidine deaminase activity in the viral lysates than the H65R mutant protein, consistent with the Western blotting analysis indicating that the P22-H65R mutant A3G was packaged into virions more efficiently than the H65R mutant A3G.

Finally, we examined whether the presence of the P22 peptide increased the ability of the CD1 domain mutants to inhibit HIV-1 replication (Fig. 7D). The P22-A3G protein was a potent inhibitor of HIV-1 replication in the absence of Vif, indicating that the presence of the P22 peptide did not interfere with the antiviral activity of the A3G protein. On the other hand, the P22-H65R protein did not inhibit HIV-1 replication, despite the fact that it was efficiently packaged into the virions. The results suggested that while the P22 peptide restored virion incorporation, it was not sufficient to overcome the defect in antiviral activity induced by the H65R mutation.

### A3G and HIV-1 Gag multimerize to reconstitute fluorescence

**Figure 8:**
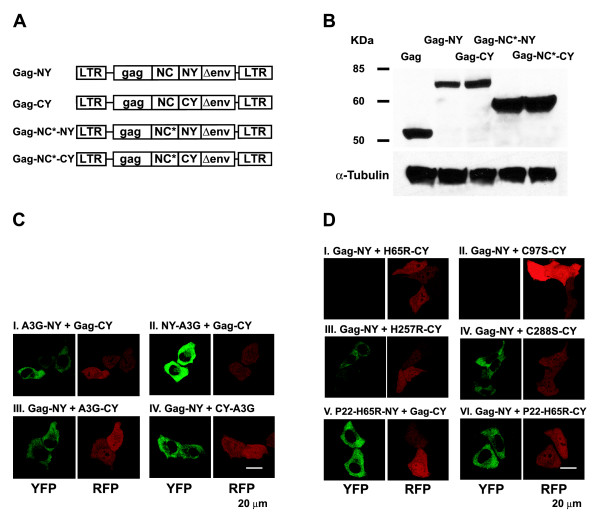
**Characterization of HIV-1 Gag BiFC constructs and interactions with A3G**. (A) Structure of HIV-1 Gag BiFC constructs. NC*, RNA-binding defective mutant of HIV-1 Gag. (B) Western blot analysis of HIV-1 Gag expression from BiFC constructs (anti-HIV-Gag polyclonal antibody) and α-tubulin in transfected 293T cells. (C) BiFC assays to evaluate interactions between HIV-1 Gag and wild-type A3G. (D) BiFC assays to evaluate interactions between HIV-1 Gag and CD1 or CD2 domain mutants of A3G.

Several studies have reported that A3G and HIV-1 Gag can be co-immunoprecipitated from cells [27-32,44]. Some studies have shown that these interactions are sensitive to treatment with RNase A, suggesting that the interactions are mediated through an RNA bridge [31,32,34], while others have reported that the co-IP is insensitive to RNase A treatment, and that A3G and the NC domain of HIV-1 Gag interact directly [28,30]. We probed the nature of A3G and HIV-1 Gag interactions in living cells by using BiFC. We used HIV-1 Gag BiFC constructs Gag-NY and Gag-CY in which either the NY or CY fragment of YFP was fused to HIV-1 Gag at its C-terminus; we also generated Gag-NC* -NY and Gag-NC*-CY constructs in which the NC domain of HIV-1 Gag contained C28H and H44C mutations in the NC zinc finger domains; mutations in the NC zinc finger domains were previously shown to significantly reduce RNA binding (Fig. 8A) [31,45-48]. Western blotting analysis of 293T cells transfected with the HIV-1 Gag expression constructs showed that all four of the HIV-1 Gag fusion proteins were expressed (Fig. 8B).

We determined the ability of the A3G fusion proteins to interact with the HIV-1 Gag fusion proteins to reconstitute fluorescence (Fig. 8C). The results showed that both A3G-NY and NY-A3G interacted with Gag-CY to reconstitute fluorescence (Fig. 8C-I and 8C-II). Similarly, both A3G-CY and CY-A3G interacted with Gag-NY to reconstitute fluorescence (Fig. 8C-III and 8C-IV).

We then sought to determine whether the CD1 and CD2 mutations in A3G influenced their ability to interact with HIV-1 Gag BiFC constructs to reconstitute YFP and fluorescence (Fig. 8D). The CD1 mutants H65R-CY and C97S-CY did not interact with Gag-NY and failed to reconstitute fluorescence (Fig. 8D-I and 8D-II), while the CD2 mutants H257R-CY and C288S-CY interacted with Gag-NY and reconstituted fluorescence (Fig. 8D-III and 8D-IV). We also asked whether the addition of the P22 peptide to the CD1 mutant H65R restored its ability to interact with HIV-1 Gag to reconstitute fluorescence. The results showed that the P22-H65R-NY + Gag-CY (Fig. 8D-V) and P22-H65R-CY + Gag-NY (Fig. 8D-VI) interacted to reconstitute fluorescence. These results suggested that A3G's ability to bind to RNA was essential and sufficient for its ability to multimerize with Gag.

**Figure 9:**
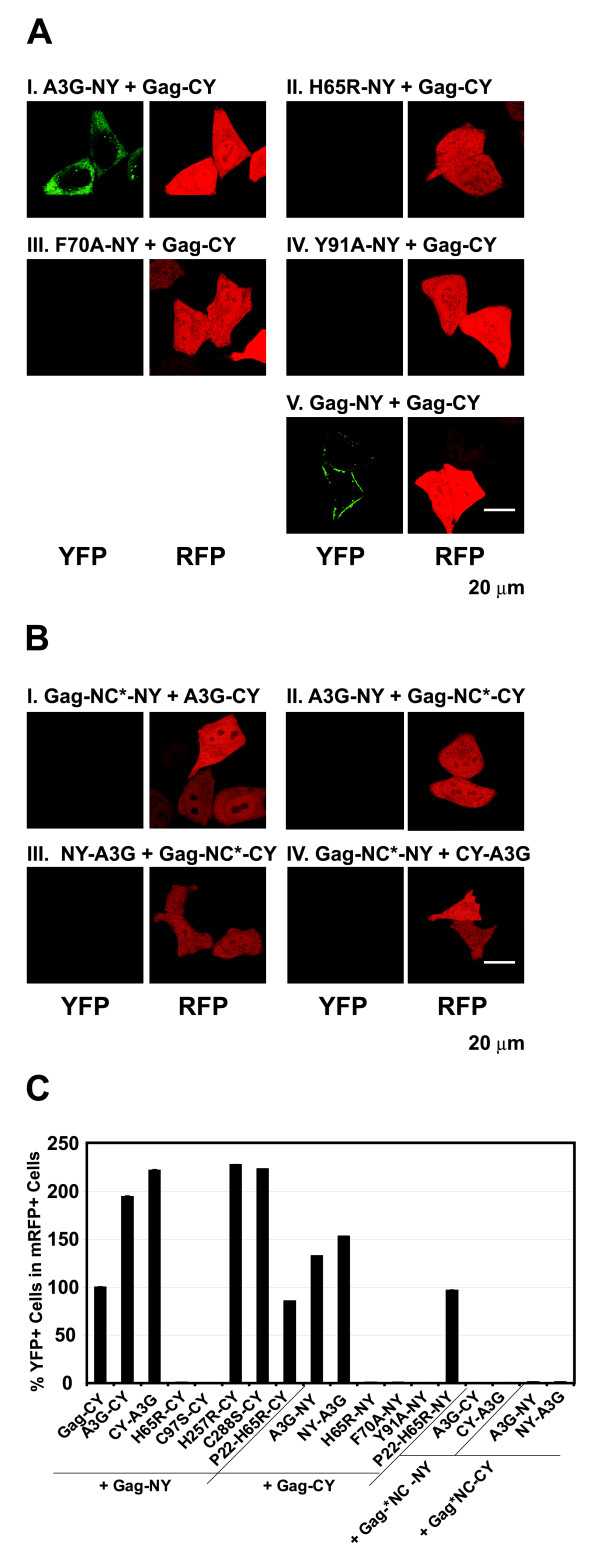
**BiFC assays to evaluate interactions between HIV-1 Gag, A3G, and RNA-binding defective mutants of Gag and A3G**. (E) BiFC assays to evaluate interactions between HIV-1 Gag and RNA-binding defective A3G mutants H65R, F70A, and Y91A. (F) BiFC assays to evaluate interactions between HIV-1 Gag NC mutants and A3G. (G) Quantification of YFP fluorescence reconstitution with HIV-1 Gag and A3G BiFC constructs using flow cytometry analysis. The percentage of YFP^+ ^cells in mRFP^+ ^cells after co-transfection with Gag-NY and Gag-CY (3.5%) was set to 100%. The error bars represent the s.e.m. of two independent experiments.

We also determined whether CD1 domain mutants that were defective in RNA binding, H65R-NY, F70A-NY and Y91A-NY could interact with Gag-CY to reconstitute YFP fluorescence (Fig. 9A). The results showed that H65R-NY (Fig. 9A-II), F70A-NY (Fig. 9A-III), or Y91A-NY (Fig. 9A-IV) did not reconstitute fluorescence with Gag-CY.

Finally, we asked whether the ability of HIV-1 Gag to bind RNA influences its ability to multimerize with A3G. Co-transfection of Gag-NC*-NY + A3G-CY, A3G-NY + Gag-NC*-CY, NY-A3G + Gag-NC*-CY, and Gag-NC*-NY + CY-A3G (Fig. 9B-I to 9B-IV) failed to reconstitute fluorescence. These results indicated that the mutations in NC, which were previously shown to impair Gag's ability to bind to RNA [31], interfered with Gag's ability to multimerize with A3G.

We performed FACS analysis to quantify the efficiency of YFP fluorescence reconstitution between Gag and A3G proteins (Fig. 9C). YFP fluorescence reconstitution with Gag-NY and Gag-CY was less efficient (~3.3%) than YFP reconstitution between A3G-NY and A3G-CY proteins (15.3%). The reason for the lower efficiency of YFP reconstitution is not known, but it may be due to virus budding and release, which result in lower steady-state levels of the Gag proteins in cells. Despite the low efficiency, the percentage of YFP^+ ^cells among mRFP^+ ^cells was significantly higher for Gag and A3G fusion proteins that were both competent to bind to RNA compared to the experiments in which one of the fusion proteins was defective in RNA binding (<0.1%).

### RNA-binding competent A3G proteins can multimerize with RNA-binding defective CD1 mutants

**Figure 10:**
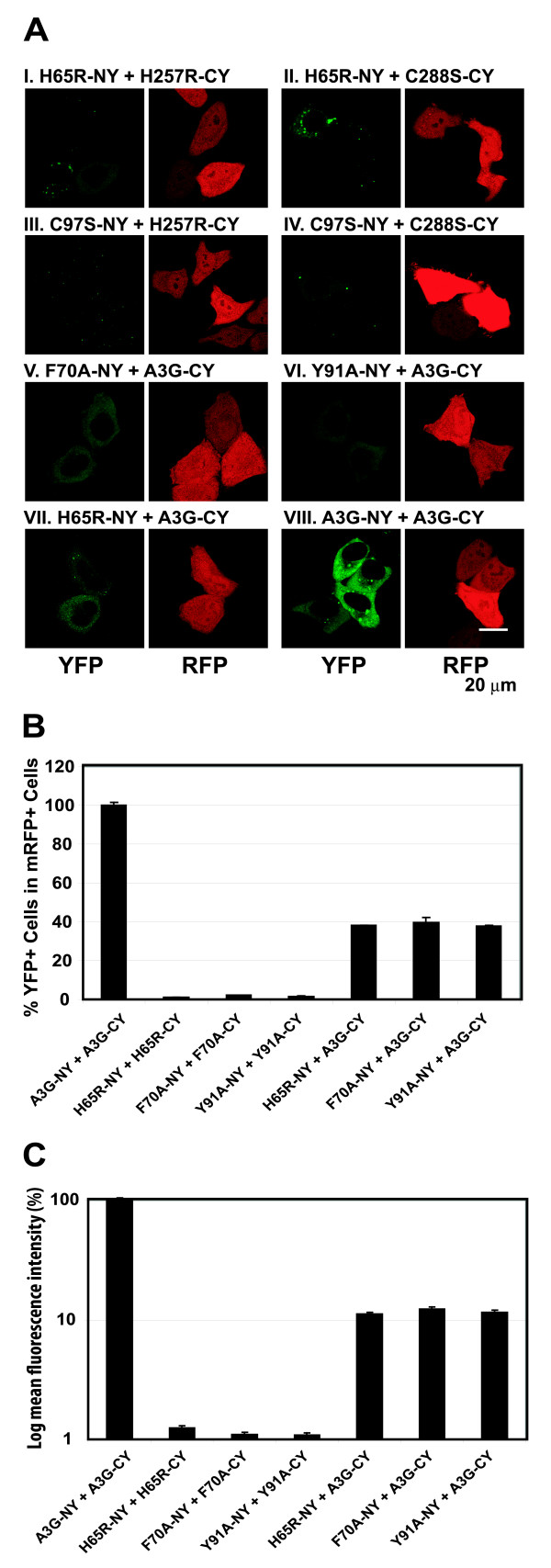
**BiFC assays to evaluate interactions between RNA-binding competent A3G proteins and RNA-binding defective A3G proteins**. (A) BiFC assays to evaluate interactions between mutant A3Gs. (B) Quantification of BiFC efficiency was determined by flow cytometry analysis as described in Fig. 2B. The percentage of YFP^+ ^cells among the mRFP^+ ^cells after cotransfection with A3G-NY and A3G-CY (15.3%) was set to 100%. The error bars represent the s.e.m. of two independent experiments. (C) Quantification of BiFC fluorescence mean intensity using flow cytometry analysis. The mean intensities of YFP^+ ^cells were determined using CELLQUEST software. The error bars represent the s.e.m. of two independent experiments.

We sought to determine whether CD1 mutant proteins can multimerize with CD2 mutants to reconstitute fluorescence. We co-transfected H65R-NY along with H257R-CY or C288S-CY (Fig. 10-I and 10-II), and C97S-NY with H257R-CY or C288S-CY (Fig. 10-III and 10-IV). Although the H65R-NY and the H65R-CY proteins failed to reconstitute fluorescence (Fig. 5B-I), they reconstituted weak fluorescence with the RNA-binding competent CD2 mutants. Similarly, the RNA-binding defective mutants F70A-NY, Y91A-NY, and H65R-NY reconstituted weak fluorescence with wild-type A3G-CY (Fig. 10-V, -VI, and 10-VII). As expected, the control wild-type A3G-NY and A3G-CY proteins reconstituted strong fluorescence (Fig. 10-VIII). These results indicated that when one of the A3G molecules binds RNA, it can interact with RNA binding defective mutants of A3G to reconstitute weak fluorescence.

The level of fluorescence reconstitution was significantly weaker when one of the A3G fusion proteins was defective in RNA binding; because of the lower level of fluorescence reconstituted, the number of YFP^+ ^cells that displayed fluorescence above the threshold of detection was also significantly lower. We quantified the efficiency of BiFC by FACS analysis to determine the percentage of mRFP^+ ^cells that were YFP^+ ^(Fig. 10B). The results showed that when A3G-NY and A3G-CY were co-expressed, YFP fluorescence was reconstituted in 95% of the mRFP-positive cells (set to 100%). When both fusion proteins were unable to bind to RNA (H65R-NY + H65R-CY, F70A-NY + F70A-CY, and Y91A-NY + Y91A-CY), less than 0.1% of the mRFP^+ ^cells were YFP^+^. As noted above, when one of the A3G fusion proteins was RNA-binding competent and the other fusion protein was RNA-binding defective (H65R-NY + A3G-CY, F70A-NY + A3G-CY, and Y91A-NY + A3G-CY), fluorescence was reconstituted in 35 – 40% of the mRFP^+ ^cells. To quantify the efficiency of BiFC reconstitution, we also compared the mean fluorescence intensity of the YFP^+ ^cells (Fig. 10C). The results showed that compared to the A3G-NY and A3G-CY positive control, the fluorescence intensity of YFP^+ ^cells was 10–15% when the cells expressed A3G-CY and RNA-binding defective mutants H65R-NY, F70A-NY or Y91A-NY. The quantification of the YFP^+ ^cells as well as their mean fluorescence intensities confirmed that the fluorescence reconstituted between wild-type A3G and RNA-binding defective mutants is weaker than that observed between two wild-type A3G molecules but stronger than that observed between two RNA-binding defective mutants.

## Discussion

In these studies, we have established a robust BiFC assay to analyze intracellular interactions between A3G and other macromolecules. We previously used the BiFC assay to demonstrate the co-assembly of HIV-1 and HIV-2 Gag proteins, and others have observed interactions between EIAV and HIV-1 Gag proteins and actin [49,50]. Our studies showed that fusion of NY or CY fragments to either the N- or C-terminus of A3G did not significantly influence A3G's ability to inhibit HIV-1 replication. Co-expression of the NY and CY fusion proteins resulted in reconstitution of BiFC, indicating that A3G formed dimers or multimers in cells.

Interestingly, fluorescence was reconstituted when the NY and CY fragments were fused to the same terminal end or at opposite terminal ends. The distance between the N- and C-termini of the full-length A3G is not known; therefore, it is unclear whether the long flexible linkers will allow reconstitution of YFP fluorescence regardless of the orientation and physical proximity of the N- and C-termini. It was recently observed that purified A3G forms tail-to-tail dimers, in which the C-terminal ends are close to each other [26,27]. Assuming that physical proximity is needed to reconstitute YFP fluorescence, our studies suggest that in the presence of RNA, head-to-head and head-to-tail dimers may also form.

### A3G multimerization is dependent on RNA binding

Our results showed that A3G multimerization was dependent on an intact CD1 while mutations in the CD2 did not influence multimerization. Co-IP assays also confirmed and extended previous observations indicating that the CD1 mutants are defective in RNA binding [25]. The observation that the CD1 mutants failed to reconstitute fluorescence in cells strongly suggested that interactions between A3G molecules in cells are dependent on RNA binding. These observations are consistent with previous biochemical studies, which showed that co-IP of A3G molecules is eliminated upon treatment of cell lysates with RNase A [22,25], and recent observations of RNA-dependent oligomerization of A3G in intracellular fluorescence resonance energy transfer [35] and co-IP assays [51]. However, another recent study observed A3G-A3G interactions in the presence of excess RNase A with wild-type A3G as well as C-terminal fragments of A3G containing the CD2 domain [27]. To exclude the possibility that C-terminal tags in our constructs interfered with RNA-independent A3G-A3G interactions, we carried out co-IP studies with untagged A3G and N-terminally tagged A3G constructs. We did not observe significant RNA-independent co-immunoprecipitations between these constructs, indicating that the C-terminal tags were not responsible for the lack of co-immunoprecipitation. The reason for the differences between our results and those of Bennett and coworkers is not clear, but may be due to differences in assay conditions.

We observed that three different RNA-binding defective mutants reconstituted weak fluorescence with wild-type A3G and with CD2 domain mutants of A3G. These observations suggest that when one A3G molecule binds RNA, it undergoes a conformational change that can permit a weak protein-protein interaction with another A3G molecule that may or may not be associated with RNA. The nature of this weak interaction is not known, but one possibility is that the weak interactions could involve previously described *in vitro *protein-protein interactions between A3G molecules and *in vivo *interactions between C-terminal halves of A3G [23,26,27]. These weak interactions were not detected in our co-IP assays (Fig. 4), suggesting that they may be transient and are not stable enough to be retained during the co-IP assays. Further studies are needed to determine the nature and functional relevance of these weak interactions. Taken together, our results are consistent with a model in which A3G molecules are monomeric in the absence of RNA and that upon binding to RNA, conformational changes allow them to interact with each other through weak protein-protein interactions.

We considered the hypothesis that the RNA binding defective mutants we tested resulted in the expression of misfolded proteins, resulting in the loss of a direct protein-protein interaction. We believe this explanation is unlikely for several reasons. First, it is unlikely that all three CD1 domain mutations (H65R, F70A, and Y91A) caused conformational changes that resulted in the loss of a direct protein-protein interaction. Second, all three mutants were sensitive to Vif-mediated proteasomal degradation, indicating that the domains of A3G involved in binding to Vif (amino acids 126–132; [52,53] were not misfolded. Third, the P22-H65R mutant of A3G exhibited cytidine deaminase activity, indicating that this mutation did not induce a conformational change in the C-terminal CD2 domain. The retention of these biological activities strongly argues that the CD1 domain mutations did not result in the expression of misfolded proteins.

### Virion incorporation of RNA-binding defective mutant A3G is restored upon fusion with non-specific RNA-binding peptide

Our studies showed that the H65R mutant of A3G was not incorporated into virions. One study found that association of A3G with 7SL RNA outside of the CD1 domain was implicated in virion incorporation of A3G [54]. However, another study reported that downregulation of 7SL RNA by overexpression of SRP19 did not influence A3G encapsidation, suggesting that 7SL RNA binding is not essential for A3G virion incorporation [55]. Other studies have found that A3G interaction with viral genomic RNA is essential for its virion encapsidation [56,57]. Our results suggest that the CD1 domain mutation either reduces A3G binding to viral or nonviral RNA [31], viral genomic RNA [56,57], or 7SL RNA [54], resulting in reduced virion incorporation. Analysis of the P22-H65R mutant showed that addition of an RNA binding domain to an RNA-binding defective mutant of A3G restored BiFC and multimerization as well as virion incorporation, but not antiviral activity. Additional studies of the P22-H65R mutant and its ability to bind to viral genomic RNA and 7SL RNA may shed light on the A3G-RNA interactions that play a critical role in A3G virion incorporation.

These results suggest that that multimers formed by an intact CD1 binding to RNA are essential for inhibition of viral replication. Multimers formed by the P22 peptide binding to RNA were nonfunctional and were unable to inhibit viral replication. Functional multimers formed by an intact CD1 binding to RNA may be essential for the previously reported processive movement on the substrate DNA [58]. Another possibility is that the CD1 and a properly formed multimer are required for A3G's incorporation into the virion core; thus, the P22-H65R multimers may be packaged but may not be associated with the reverse transcription complex in the virion cores. This hypothesis is consistent with a recent report that a fusion protein between the CD1 of A3G and APOBEC3A allowed APOBEC3A to be associated with the nucleoprotein complexes in the viral core; furthermore, the A3G-A3A fusion protein inhibited viral replication [59]. Additional biochemical studies are needed to determine why the P22-H65R mutant is packaged but fails to inhibit HIV-1 replication.

### Association of A3G with HIV-1 Gag is dependent on RNA binding

RNA-binding defective A3G mutants failed to reconstitute BiFC with HIV-1 Gag constructs and an RNA-binding defective HIV-1 Gag NC mutant also did not reconstitute BiFC with wild-type A3G, suggesting that interaction between Gag and A3G are mediated by an RNA bridge. This observation is consistent with our previous results that co-IP of Gag with A3G was abolished by treatment of the cell lysate with RNase A [31]. However, additional protein-protein interactions between A3G and HIV-1 Gag upon binding to RNA cannot be excluded. Previous studies showed that the NC mutant pCCHH (C28H/H44C) packaged very little HIV-1 RNA (<5%) and very low amounts of non-specific RNA (~20%) [31,45]. *In vitro *studies have suggested that mutations in the zinc finger domains of NC have a lower affinity for RNA [45-48]. Our studies suggest that the double mutant is severely defective in RNA binding, which results in the reduced packaging of non-specific RNA [31].

The basic linker region of HIV-1 NC, and a peptide motif at the C-terminal end of the NC domain of human T cell leukemia virus type 1 (HTLV-1) have been implicated in promoting and inhibiting virion incorporation of A3G, respectively [35,60]. Our results suggest that an intact structure of the HIV-1 NC zinc-binding domain influences RNA binding by HIV-1 Gag, which indirectly influences A3G virion incorporation.

The BiFC approach provides an alternative to the FRET assay that was recently described [35]. In practice, the BiFC assay is free from background fluorescence since the NY and CY fragments of YFP do not fluoresce. On the other hand, the FRET assay provides an ability to quantify the efficiency of interactions between binding partners [35]. These studies provide a powerful approach and novel insights into the *in vivo *assembly of A3G multimers, A3G-Gag interactions, and virion incorporation of A3G.

## Conclusion

We have developed a robust BiFC assay to analyze intracellular A3G-A3G, A3G-Gag, and A3G-RNA interactions. These studies show that A3G forms multimers in cells and that this multimerization is dependent on RNA binding. A heterologous RNA binding motif restored multimerization but not antiviral activity, suggesting that formation of functional multimers is dependent on an intact CD1 that is capable of RNA binding. Finally, we observed weak interactions between an A3G molecule with an intact CD1 and another A3G that is defective in RNA binding. The weak interactions could explain previously described protein-protein interactions between purified A3G molecules.

## Methods

### Plasmids and their construction

HIV-1-based vector pHDV-EGFP was kindly provided by Derya Unutmaz (New York University) [61]; pHCMV-G expresses the vesiculostomatitis virus envelope glycoprotein G from a human cytomegalovirus promoter [62]. pC-Help*ΔVif*, kindly provided by Klaus Strebel (National Institute of Allergy and Infectious Diseases, National Institutes of Health, Bethesda, MD), is a derivative of pC-Help; it is an HIV-1 helper construct that lacks several *cis*-acting elements needed for viral replication, including the packaging signal and primer-binding site. pC-Help*ΔVif *expresses all of the viral proteins except Vif, Nef, and Env [63].

To generate A3G-NY and A3G-CY, A3G sequences were amplified by PCR and replaced the gag sequences of previously described HIV-1-gag-NY and HIV-1-gag-CY [49]. The resulting plasmids were digested with *Nhe*I and *BsiW*I and the 1773 and 1455 bp fragments containing A3G and NY or CY, respectively, were cloned into the *Nhe*I and *Acc65*I restriction sites of pCR 3.1 vector (Invitrogen) to generate A3G-NY and A3G-CY. In these constructs, the A3G and the NY (1–172 amino acids of YFP) or CY (173–238 amino acids of YFP) sequences are separated by a 12 amino acid glycine-rich hinge sequence (PGISGGGGGILD) to provide flexibility to the fusion protein.

To generate NY-A3G and CY-A3G, NY and CY fragments were amplified using HIV-1-gag-NY and HIV-1-gag-CY as templates and subcloned into pcDNA-EYFP-A3G vector, a derivative of pcDNA-APO3G. The NY and CY fragments and A3G in the NY-A3G and CY-A3G constructs are separated by a 19 amino acid glycine-rich flexible sequence (EGITGGGGGILDGYLQNSR) to provide flexibility to fusion proteins.

A3G BiFC constructs containing H65R, C97S, F70A, Y91A, H257R, or C288S mutations were generated by the QuikChange Mutagenesis Kit (Stratagene). The presence of the desired mutations and the absence of inadvertant mutations were verified by DNA sequencing.

P22-H65R-NY was constructed by inserting a synthetic double-stranded DNA fragment encoding P22 peptide into H65R-CY to generate P22-H65R-CY. The CY fragment of P22-H65R-CY was replaced with the NY fragment to generate P22-H65R-NY. The plasmids P22-A3G and P22-H65R were constructed in a similar manner.

HIV-1-gag-NY and HIV-1-gag-CY plasmids have been described previously [49]. Gag-NC* -NY was generated by replacing the NC domain of the wild-type HIV-1 Gag with NC zinc finger binding domain mutant CCHH/CCCC, which contains C28H and H44C substitutions [45]. The sequences and structures of all constructs were determined by DNA sequencing.

### Transfections, infections, and flow cytometry analysis

Human 293T cells were maintained as described previously [31] and transfected by using a FuGENE 6 Transfection Reagent (Roche) or by the calcium phosphate precipitation method [64]. Virus was harvested 48 hours post-transfection, and the p24 capsid amounts in the culture supernatants were determined by ELISA (Perkin-Elmer). The 293T cells were infected with virus preparations containing 100 ng of p24 capsid. The infected cells were analyzed by flow cytometry (FACScan, Becton Dickinson) for green fluorescence 48 hours after infection, and the results were analyzed with CELLQUEST software (Becton Dickinson). To determine the effects of A3G or A3G-derived constructs on HIV-1 replication in the absence of Vif, pHDV-EGFP, pC-Help*ΔVif*, pHCMV-G, and pcDNA-A3G or A3G-derived constructs were co-transfected by using 1:0.8: 0.25:0.25 μg of DNA per 35-mm-plate, respectively. The molar ratios of pHDV-EGFP, pC-Help*ΔVif*, pHCMV-G, and the pcDNA-A3G or A3G-derived constructs were approximately 1:1:0.4:0.4, respectively. Flow cytometry analysis was also used to quantify YFP complementation from transiently transfected 293T cells.

### Cytidine deamination assay and western blotting analysis

Total cellular protein extracts were prepared as described [65], protein concentrations were determined using the Bradford protein assay (Bio-Rad Laboratories), and 0.3 μg of the protein was used to determine cytidine deamination activity using a previously described scintillation proximity assay for cytidine deamination [31].

For Western blotting analysis, the 293T cells were co-transfected with A3G-expressing plasmids and pC-Help*ΔVif *or phVif. Approximately 48 hours after transfection, 2 × 10^6 ^cells were harvested, washed twice with ice-cold phosphate-buffered saline (PBS), and lysed in 1 ml of Complete M-Lysis Buffer in the presence of protease inhibitor (Roche). Cell extracts were adjusted to equivalent protein concentration, and equal aliquots were used for western blotting analysis. A3G proteins were detected by using ApoC17 rabbit anti-human A3G antiserum (a kind gift from Klaus Strebel) or anti-c-Myc antibody produced in rabbit (Sigma-Aldrich). Vif proteins were detected using a rabbit anti-Vif polyclonal antibody at a 1:5000 dilution [10], followed by an HRP-conjugated, stabilized goat anti-rabbit secondary antibody (Pierce) at a 1:10,000 dilution. The tubulin protein was detected by using the anti-tubulin antibody (Sigma-Aldrich). Viral like particles (VLPs) were produced from 293T cells transfected with pHDV-EGFP, pC-Help*ΔVif*, pHCMV-G as well as pcDNA-A3G, or A3G-derived constructs, or both HIV-1 and A3G-expressing plasmids by using a calcium phosphate precipitation method. VLPs were harvested 48 hours after transfection, filtered using 0.45-μm membranes (Nalgene), and concentrated 20-fold by ultracentrifugation as described previously [10]. Viral lysates containing 100 ng of p24 CA of 293T-derived HDV-GFP Δ*vif *virions were loaded per lane. HIV-1 Gag, Gag-NY, Gag-CY, and Gag-NC*-NY proteins were detected using human polyclonal HIV immunoglobulin (HIV-IG) antiserum (AIDS Research and Reference Reagent Program, Division of AIDS, NIAID, NIH).

Quantfication of the Western blots was performed using the Odyssey software version 2.1 (Licor, Inc.). In addition, SuperSignal West Femto Maximum secondary antibody and Sensitivity Substrate (Pierce) was used in some Western blotting experiments.

### BiFC assay

The BiFC assay to examine the interactions were performed by using an adaptation of a previously described procedure [36,37]. HeLa cells were plated on glass bottom No 1.5 uncoated γ-irradiated plates (MatTek Corp., Ashland, MA) 24 hours prior to transfection and maintained in phenol-red free Dulbecco modified Eagles' medium (Mediatch, Inc., Herndon, VA) supplemented with L-glutamine and 10% fetal calf serum. HeLa cells were transfected with 0.25 μg of A3G BiFC constructs per 35-mm dish, unless stated otherwise; in experiments designed to analyze interactions with HIV-1 Gag, 0.5 μg of A3G and 0.5 μg of HIV-1 Gag BiFC constructs were co-transfected with 0.075 μg of mRFP1, unless stated otherwise. The growth media was changed 6 hours before and after transfection. Live cell imaging was performed at 37°C and 5% CO_2 _after approximately 14 hours post-transfection using Zeiss LSM510 META confocal fluorescence microscope (Carl Zeiss) and 40× NA 1.3 oil immersion objective. A 488-nm argon laser with a 500- to 530-nm band-pass emission filter and a 543-nm helium-neon laser with a 615- to 675-nm band-pass emission filter were used.

In order to quantify YFP fluorescence complementation, 293T cells were transfected with 0.25 μg of each BiFC constructs per 35-mm dish, and 0.15 μg mRFP1 plasmid was co-transfected per transfection as an internal control. The transfected cells were harvested approximately 14 hours post-transfection. Cells were washed twice with 1 ml of filtered 1× PBS, resuspended in 500 μl 1× filtered PBS, and fixed with 100 μl of 6% paraformaldehyde for two hours. The fixed cells were filtered using a 5 ml Polystrene round-bottom tube with a cell-strainer cap (12 × 17 mm style; BD Falcon) before flow cytometry analysis. The transfected cells were analyzed by flow cytometry according to the method described above. To quantify the amount of complementation we analyzed both YFP and mRFP positive cells and determined the percentage of mRFP^+ ^cells that were YFP^+^.

### Co-IP and protein visualization

293T cells were seeded at 4 × 10^6 ^cells per 100-mm dish and were co-transfected 24 hours later using FuGene 6 Transfection Reagent (Roche) with 1 μg of each pF-A3G and pcDNA3.1noMCS (empty vector), 1 μg each of pF-A3G and A3G-CY, 1 μg each of pF-A3G and P22-H65R or P22-H65R-CY, or 1 μg of pF-A3G and 4 μg each of H65R-CY or F70A-CY (Roche). The supernatant was removed 48 hours post-transfection, and the cells were washed twice in 10 ml of phosphate buffered saline (PBS). The cells were then lysed in 1 ml Complete M-Lysis Buffer in the presence of protease inhibitor (Roche), by incubation with gentle agitation for 15 min. The cellular debris was removed by centrifugation at 10,000 × g for 10 minutes, and the resulting supernatant was divided into two tubes each contained 500 μl of the cell lysate. The lysate was treated with 100 μg/ml RNase A or 80 U/μl of RNase inhibitor (RNaseOUT; Invitrogen). These lysates were incubated at 37°C for 1 hour. We then added to the cell lysates 80 μl of 1× IP buffer (Sigma) and 20 μl of an anti-FLAG M2 agarose affinity gel or anti-c-Myc affinity gel (Sigma). The FLAG- or c-Myc-tagged A3G proteins were allowed to bind to the agarose affinity gel by gentle rotation at 4°C overnight, after which any unbound proteins were removed by washing six times using 1× IP buffer and a final wash with 0.1× IP buffer. The bound protein complexes were then eluted using a 1× Laemmli sample buffer (Sigma). The eluted complexes, as well as the input cell lysates, were then analyzed by polyacrylamide gel electrophoresis and Western blotting. Untagged A3G and FLAG- or c-Myc tagged A3G proteins were detected by using ApoC17 rabbit anti-human A3G antiserum, a rabbit anti-FLAG polyclonal antibody (Sigma), or a rabbit anti-c-Myc monoclonal antibody (Sigma) at a 1:5,000 dilution, followed by a HRP-labeled goat anti-rabbit secondary antibody (Sigma) at a 1:10,000 dilution. The proteins were visualized using a Western Lighting Chemiluminescence Reagent Plus kit (PerkinElmer).

### Immunofluorescence staining of transfected cells

HeLa cells were plated on 15-mm-diameter coverslips (BD Biosciences) for 24 hours prior to transfection. At 15 hours post-transfection, cells were fixed for 20 minutes at room temperature in 5% paraformadehyde and 2% sucrose in PBS, and permeabilized for 30 minutes with 1% Triton X-100 and 10% sucrose in PBS. Anti-A3G polyclonal antiserum was used as a primary antibody and Alexa Fluor 488-conjugated goat antibody to rabbit IgG (H+L) (Molecular Probes) as secondary antibody. Cells were incubated sequentially with each antibody for 1 hour and washed three times. All washes and antibody dilutions were performed in PBS containing 1% bovine serum albumin (Sigma), 1% Triton X-100 (Sigma), and 2% normal goat serum (Vector Laboratories, Inc.). Finally, the nuclei were stained by incubating the cells in PBS containing 2 μg/ml of 4', 6-diamidino-2-phenyl-indole (DAPI; Sigma) for 2 minutes followed by two washes in PBS. The coverslips were rinsed in deionized water and then mounted on glass slides by using FluoroGuard (Bio-Rad). The cells were visualized using a LSM510 Zeiss confocal fluorescence microscope (Carl Zeiss).

## Abbreviations

HIV-1: human immunodeficiency virus type 1; Vif: viral infectivity factor; APOBEC3G and A3G: apolipoprotein B mRNA-editing enzyme catalytic polypeptide-like 3G; CD1 and CD2: catalytic domains 1 and 2 of A3G; BiFC: bimolecular fluorescence complementation; YFP: yellow fluorescent protein; NY: N-terminal 1–172 amino acids of YFP; CY: C-terminal 173–238 amino acids of YFP.

## Competing interests

The authors declare that they have no competing interests.

## Authors' contributions

YNF performed most of the experiments. VB constructed and analyzed HIV-1 Gag BiFC expression plasmids. WSH provided intellectual input in the design and analysis of the experiments. VKP supervised and directed the studies and data analysis. All authors approved and contributed to the preparation of the final manuscript.
